# Induction of interleukin-10 is dependent on p38 mitogen-activated protein kinase pathway in macrophages infected with porcine reproductive and respiratory syndrome virus

**DOI:** 10.1186/1743-422X-9-165

**Published:** 2012-08-21

**Authors:** Jun Hou, Lianghai Wang, Rong Quan, Yi Fu, Hexiao Zhang, Wen-hai Feng

**Affiliations:** 1 State Key Laboratories of Agrobiotechnology, Department of Microbiology and Immunology, College of Biological Science, China Agricultural University, Beijing, 100193, China; 2 Department of Microbiology and Immunology, College of Biological Science, China Agricultural University, Beijing, 100193, China; 3 Present address: Shihezi University School of Medicine, Shihezi, Xinjiang, 832002, China; 4 Beijing Entry-exit inspection and quarantine bureau, No.6 Tian shui yuan road, Beijing, 100026, China

## Abstract

**Background:**

Porcine reproductive and respiratory syndrome virus (PRRSV) causes reproductive failure and respiratory illness in pigs and usually establishes a persistent infection. Previous studies suggested that interleukin-10 (IL-10) could play a critical role in PRRSV-induced immunosuppression. However, the ability of PRRSV to induce IL-10 in infected cells is controversial. In this study, we further investigated this issue using PRRSV strain CH-1a, which is the first North American genotype strain isolated in China.

**Results:**

PRRSV strain CH-1a could significantly up-regulate IL-10 production both at mRNA and protein levels in porcine alveolar macrophages (PAMs), bone marrow-derived macrophages (BMDMs), and monocyte-derived macrophages (MDMs). However, up-regulation of IL-10 by PRRSV was retarded by specific inhibitors of p38 mitogen-activated protein kinase (MAPK) (SB203580) and NF-κB (BAY11-7082). Additionally, p38 MAPK and NF-κB pathways but not ERK1/2 MAPK were actually activated in PRRSV-infected BMDMs as demonstrated by western blot analysis, suggesting that p38 MAPK and NF-κB pathways are involved in the induction of IL-10 by PRRSV infection. Transfection of PAMs and PAM cell line 3D4/21 (CRL-2843) with viral structural genes showed that glycoprotein5 (GP5) could significantly up-regulate IL-10 production, which was dependent on p38 MAPK and signal transducer and activator of transcription-3 (STAT3) activation. We also demonstrated that a full-length glycoprotein was essential for GP5 to induce IL-10 production.

**Conclusions:**

PRRSV strain CH-1a could significantly up-regulate IL-10 production through p38 MAPK activation.

## Background

Porcine reproductive and respiratory syndrome (PRRS) is characterized by respiratory disease in piglets and severe reproductive failure such as a high rate of late term abortion and early farrowing in sows [1-3]. The etiologic agent is PRRS virus (PRRSV), which contains 10 open reading frames (ORFs) that encode 14 non-structural proteins (NSPs) and 8 structural proteins. ORFs 2–7 are located in the 3’terminal region of the genome and encode structural proteins including the minor envelope glycoproteins GP2 (ORF2a), GP3 (ORF3), GP4 (ORF4), small hydrophobic proteins E (ORF2b) and the recently discovered ORF5a protein, the major envelope glycoprotein GP5 (ORF5), the non-glycosylated membrane protein M (ORF6), and the nucleocapsid protein N (ORF7) [4-7]. PRRSV has two genotypes, the European genotype (type I) and North American genotype (type II), according to phylogenetic analysis [8].

Pigs that survive from the acute stage of PRRSV infection usually develop persistent infection up to 150 days [9], which is probably due to the weak immune responses such as poor interferon alpha (IFN-α) production [10], delayed and weak neutralizing antibody response [11,12], and lower T cell mediated immune response [13]. Extensive studies have been showing that many intracellular pathogens that specifically target macrophages for infection could exploit IL-10 to suppress host innate and adaptive immune responses [14-17]. The mRNA profiles in bronchoalveolar lavage cells (BALC) from piglets infected *in-utero* with PRRSV suggested that IL-10 could play a role in PRRSV-induced immunosuppression [18]. Previous studies also showed that PRRSV infection *in vitro* significantly up-regulated IL-10 gene expression in porcine peripheral blood mononuclear cells (PBMC), porcine alveolar macrophages (PAMs), bone marrow-derived immature dendritic cells (BM-imDCs), and PBMC-derived mature dendritic cells [19-22]. In *in-vivo* model, both the European and North American PRRSV strains could significantly induce IL-10 gene expression in PBMC and BALC of infected pigs [19]. However, a recent study showed that there were differences among various European strains of PRRSV in IL-10 induction in DCs [23]. In the case of North American PRRSV strains, SD-23983 lacked the capacity to up-regulate IL-10 in DCs [24]. A virulent strain vFL12, which is derived from an infectious cDNA clone, also could not up-regulate IL-10 expression both *in vitro* and *in vivo*[25]. Therefore, the ability of PRRSV to induce IL-10 production could be strain-dependent and needs to be further investigated.

IL-10 gene expression in macrophages can be induced by stimuli such as lipopolysaccharide (LPS). It has been demonstrated that LPS-induced IL-10 production in human peripheral blood monocytes is dependent on the endogenous pro-inflammatory cytokines IL-1 and/or tumor necrosis factor alpha (TNF-α) through p38 but not ERK1/2 mitogen-activated protein kinase (MAPK) signaling pathway activation [26]. Whereas stimulation of murine bone marrow-derived macrophages (BMDMs) with LPS plus FcgammaR (FcγR) ligation leads to enhanced ERK1/2 activation and increased signal transducer and activator of transcription-3 (STAT3) binding to the IL-10 promoter [27]. Zymosan, a stimulus for Toll-like receptor TLR2 and dectin-1, induces DCs to secrete abundant IL-10 through activation of ERK1/2 [28]. However, the regulation of IL-10 production in porcine macrophages during PRRSV infection is poorly understood. In the present work, we investigated the ability of PRRSV to induce IL-10 production in porcine macrophages and its underlining molecular mechanisms.

## Results

### IL-10 production is up-regulated at both mRNA and protein levels after PRRSV infection

**Figure 1:**
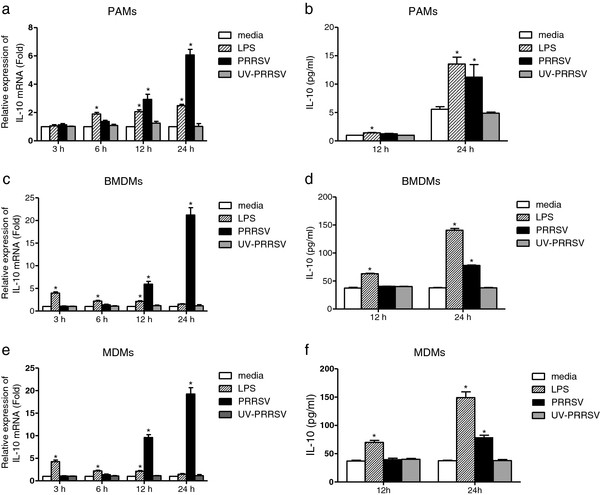
**IL-10 was up-regulated in PRRSV-infected macrophages.** PAMs and BMDMs were infected with PRRSV or UV-inactivated virus at an MOI of 1. Cells stimulated with LPS (1 μg ml^-1^) were used as positive control. (**a**), (**c**), (**e**) Cells were harvested at the indicated time points and real-time PCR was performed to evaluate IL-10 mRNA levels. (**b**), (**d**), (**f**) Measurement of secreted IL-10 protein levels in the supernatant of PRRSV-infected PAMs and BMDMs at indicated time points by ELISA. The results represent means ± SD of three independent experiments. *Significant difference (P < 0.05) from media control using *Student’s t-test*.

Previous studies have shown that different PRRSV isolates and genotypes might have a distinct ability to induce IL-10 production in infected cells. We further investigated this issue using PRRSV strain CH-1a, which is the first North American genotype strain isolated in China in 1996. PAMs, BMDMs, and monocyte-derived macrophages (MDMs) were inoculated with PRRSV or UV-inactivated virus. IL-10 mRNA was significantly up-regulated in PAMS at 12 h (2.9-fold) and 24 h (6.1-fold) post PRRSV infection (h.p.i.) (Figure 1a). Secreted IL-10 protein level was also increased at 24 h.p.i. (Figure 1b). In addition, PRRSV infection induced a higher expression of IL-10 in BMDMs compared with PAMs both at the transcriptional level (about a 21-fold increase in BMDMs and a 6-fold increase in PAMs at 24 h.p.i.) and translational level (about 78 pg ml^-1^ in BMDMs and 12 pg ml^-1^ in PAMs at 24 h.p.i.) (Figure 1c and 1d). The results in MDMs were nearly the same as in BMDMs (Figure 1e and 1f). However, the UV-inactivated virus did not significantly induce IL-10 production either at IL-10 mRNA level or at protein level. Thus, these results suggested that the PRRSV strain CH-1a could stimulate IL-10 production in PAMs, BMDMs, and MDMs *in vitro*.

### Inhibition of p38 MAPK and NF-κB pathways abrogates the production of IL-10

**Figure 2:**
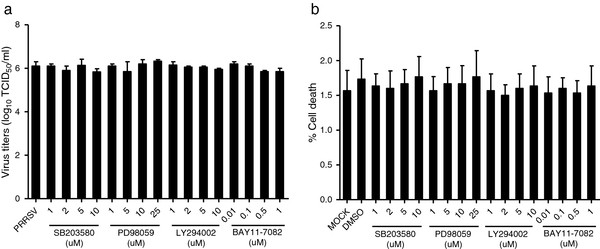
**Viral replication was not obviously affected when cells were treated with inhibitors of p38 (SB203580), ERK1/2 (PD98059), PI3K (LY294002), and NF-κB (BAY11-7082) pathways.** (**a**) BMDMs were pretreated with DMSO or PI3K inhibitor LY294002, ERK MAPK inhibitor PD98059, p38 inhibitor SB203580, and NF-κB inhibitor BAY11-7082 at the indicated concentrations for 2 h. Cells were then infected with PRRSV (MOI = 1) and harvested at 24 h.p.i.. Virus titers in the cell supernatants were measured by a standard 50% tissue culture infective doses (TCID_50_) assay. (**b**) BMDMs were treated with DMSO or PI3K inhibitor LY294002, ERK MAPK inhibitor PD98059, p38 inhibitor SB203580, and NF-κB inhibitor BAY11-7082 at the indicated concentrations for 24 h. Then the cytotoxicity of the inhibitors on BMDMs was determined by trypan blue exclusion dye staining. Data represent means ± SD of three independent experiments.

We first examined the effect of signal transduction pathway inhibitors on PRRSV replication. BMDMs were infected with PRRSV in the presence of either the PI3K inhibitor LY294002, the ERK1/2 inhibitor PD98059, the p38 inhibitor SB203580, or the NF-κB inhibitor BAY11-7082 at various concentrations, and the virus titers in the cell supernatants at 24 h.p.i were determined. The cytotoxicity of all the inhibitors was determined by trypan blue exclusion dye staining. All concentrations of the inhibitors used in this study neither caused detectable cell death nor significantly altered PRRSV replication in BMDMs (Figure 2).

**Figure 3:**
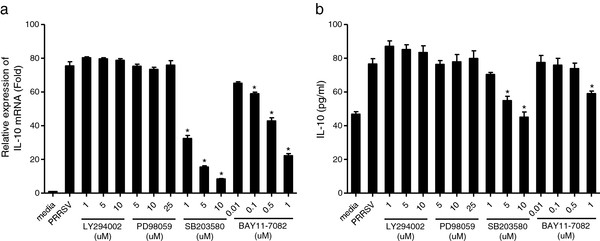
**PRRSV-stimulated IL-10 production was selectively inhibited by inhibitors of p38 MAPK and NF-κB signaling pathways.** BMDMs were pretreated with DMSO or PI3K inhibitor LY294002, ERK MAPK inhibitor PD98059, p38 inhibitor SB203580, and NF-κB inhibitor BAY11-7082 at the indicated concentrations for 2 h. Cells were then infected with PRRSV (MOI = 1) and harvested at 24 h.p.i.. (**a**) Total RNA isolated from cells was reverse transcribed and then analyzed using real-time PCR. (**b**) IL-10 production in cell supernatants was analyzed by ELISA. Data represent means ± SD of three independent experiments. *Significant difference (P < 0.05) from PRRSV control using *Student’s t-test*.

Then, we investigated which signaling pathway was involved in the regulation of IL-10 production by PRRSV infection. Our results showed that IL-10 mRNA expression in BMDMs was significantly decreased to about 43%, 21%, and 11% when treated with p38 inhibitor SB203580 at 1 μM, 5 μM, and 10 μM, respectively (Figure 3a). NF-κB inhibitor BAY11-7082 aslo significantly inhibited IL-10 expression in BMDMs and the IL-10 level was dose-dependently down to 86%, 78%, 57%, and 29% when compared with the PRRSV infected control (Figure 3a). PRRSV induced IL-10 production at the protein level was also significantly decreased when treated with high concentration of p38 MAPK (5 μM and 10 μM) and NF-κB inhibitors (1 μM) (Figure 3b). Neither PI3K inhibitor LY294002 nor the ERK1/2 inhibitor PD98059 inhibited PRRSV-induced IL-10 production. Similar results were also observed in p38 and NF-κB pathway inhibitor treated PAMs (data not shown). These results suggested that p38 MAPK and NF-κB pathways might be involved in PRRSV-induced IL-10 production.

### PRRSV-infection activates p38 MAPK and NF-κB pathway in BMDMs

**Figure 4:**
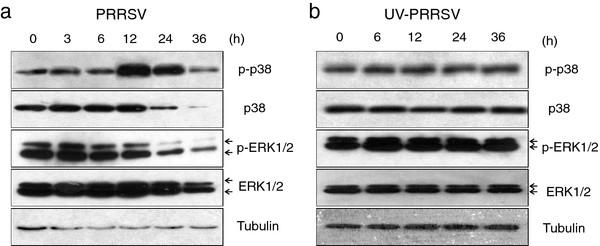
**PRRSV infection activated p38 MAPK signaling pathway in BMDMs.** Whole-cell extracts of BMDMs infected with (**a**) PRRSV at an MOI of 1 or (**b**) equal amounts of UV-inactivated virus were prepared at indicated time points and analyzed by immunoblot for the phosphorylation status of p38 (p-p38) and ERK1/2 (p-ERK1/2). The blot was also probed for tubulin as loading control.

To confirm that p38 MAPK and NF-κB signaling pathways are activated in PRRSV-infected BMDMs, p38 and ERK1/2 phosphorylation and IκB degradation were analyzed by western blot. As shown in Figure 4(a), the phosphorylated p38 was significantly increased at 12 h and 24 h post infection. PRRSV infection slightly stimulated ERK1/2 phosphorylation in BMDMs at 3 h.p.i., and then the phosphorylation gradually declined. At 36 h.p.i., viral infection induced obvious cytopathic effect (CPE) which may lead to the decreases of p38 and ERK1/2. Cells inoculated with UV-irradiated virus did not enhance p38 and ERK1/2 phosphorylation (Figure 4b). Furthermore, IκB was gradually degraded in PRRSV-infected BMDMs [29], MARC-145, and PAMs [30,31]. Thus, these results suggested that PRRSV infection significantly activated the p38 MAPK and NF-κB pathways but not ERK1/2 MAPK pathway.

### GP5 induces IL-10 production both at mRNA and protein levels

**Figure 5:**
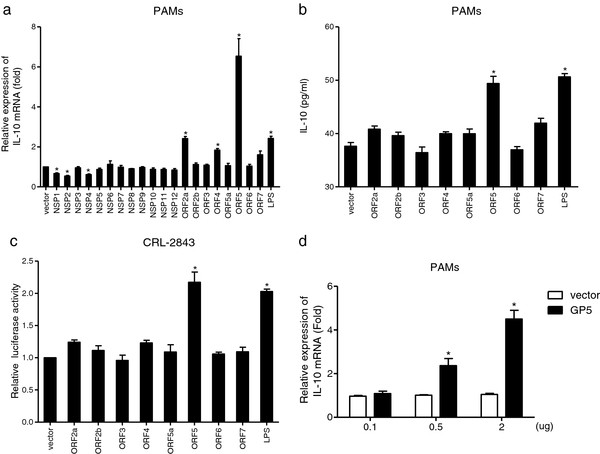
**GP5 induced IL-10 mRNA and protein expression.** (**a**) PAMs were transfected with PRRSV structural and non-structural proteins (2 μg) or stimulated with LPS (1 μg ml^-1^) as positive control. IL-10 mRNA expression at 24 h post transfection was evaluated by real-time PCR. (**b**) Cell supernatants collected from viral structural protein transfected PAMs were analyzed for IL-10 production by ELISA. (**c**) CRL-2843 cells were cotransfected with a mixture of pIL-10-luc plasmid, pRL-TK plasmid, and viral structural protein-encoding plasmids or empty vector. At 24 h post transfection, luciferase activities were measured with the Dual-Luciferase Reporter Assay System. (**d**) Increasing amounts (0.1, 0.5 and 2 μg) of GP5-encoding plasmid were transfected into PAMs. IL-10 mRNA expression at 24 h post transfection was examined by real-time PCR. The results represent means ± SD of three independent transfections. * Significant difference (P < 0.05) from empty vector control using *Student’s t-test*.

To examine which PRRSV protein could induce IL-10 production, all of the PRRSV structural and non-structural protein genes (derived from the viral genome of CH-1a strain) were cloned into a mammalian expression vector and verified for expression, except for NSP6 which has only 16 amino acids. Each of these constructs was transfected into PAMs and real-time PCR was performed. As shown in Figure 5(a), IL-10 gene expression was significantly induced by ORF2a, ORF4 and ORF5 compared with empty vector (2.4-fold, 1.8-fold, and 6.5-fold, respectively). On the contrary, none of the PRRSV NSPs could induce IL-10 mRNA production and among all NSPs screened, NSP1, NSP2, and NSP4 down-regulated IL-10 mRNA levels to 68%, 54%, and 61% compared with control vector, respectively. Similar results were obtained in the PAM cell line 3D4/21 (CRL-2843) (data not shown). Among the PRRSV structural proteins, only GP5 significantly induced IL-10 production in PAMs at protein level when compared to controls (Figure 5b), which was further confirmed with the result that porcine IL-10 promoter activity was increased to about 2.2-fold in GP5- transfected CRL-2843 cells (Figure 5c). In addition, our results showed that induction of IL-10 by PRRSV GP5 was in a dose-dependent manner (Figure 5d).

### The intact GP5 protein is required for the induction of IL-10

**Figure 6:**
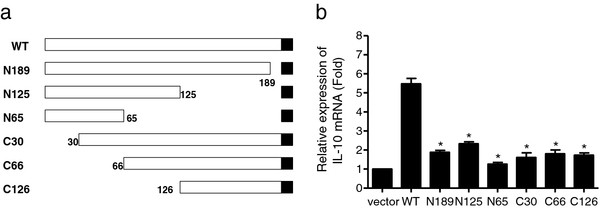
**The full length of GP5 was required for IL-10 induction.** (**a**) Schematic representation of truncated GP5 (numbers indicate the amino acids positions). The black squares represent the translation termination. (**b**) PAMs were transfected with the truncated GP5 expression plasmids (2 μg) or empty vector. Cells were harvested at 24 h post transfection. Total RNA isolated from cells was reverse transcribed and real-time PCR was performed to detect porcine IL-10 mRNA. Results are shown as means ± SD of three independent transfections. *Significant difference (P < 0.05) from wild-type GP5 using *Student’s t-test*.

To determine the essential region of GP5 to induce IL-10 production, we transiently transfected PAMs with constructs encoding wild-type GP5 or C-terminal and N-terminal deletion mutants (Figure 6a). As shown in Figure 6(b), the ability of deletion constructs N189, N125, C30, C66, and C126 to induce IL-10 expression was dramatically decreased compared with wild-type GP5. Moreover, IL-10 expression induced by a C-terminal-deletion construct N65 was down to the basal level. These results suggested that the intact GP5 was required for its ability to induce IL-10 expression.

### p38 MAPK and STAT3 pathways are involved in GP5-induced up-regulation of IL-10

**Figure 7:**
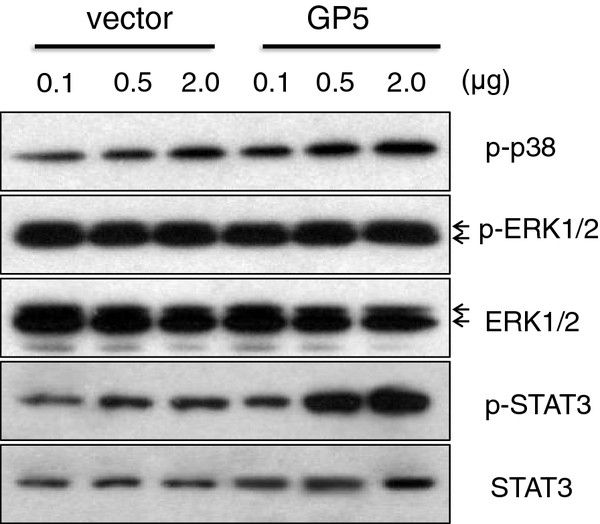
**GP5 expression activated p38 MAPK and STAT3 pathways.** At 24 h post transfection of the vector control or GP5-encoding plasmid into CRL-2843 cells, the phosphorylation status of p38 (p-p38), ERK1/2 (p-ERK1/2) and STAT3 (p-STAT3) were evaluated by western blot.

It was of interest to determine which pathways were involved in GP5-induced IL-10 production. As shown in Figure 7, GP5 dose-dependently (0.1, 0.5, and 2 μg) activated p38 MAPK but not ERK1/2. STAT3 was also activated in GP5 transfected CRL-2843 cells. Collectively, these results indicated that p38 MAPK and STAT3 pathways were involved in GP5-induced production of IL-10.

## Discussion

IL-10 is a regulatory cytokine which is known to inhibit production of several pro-inflammatory cytokines such as IL-1 and TNF-α. In this study, we showed that IL-10 was up-regulated in PRRSV CH-1a-infected PAMs, BMDMs, and MDMs both at mRNA and protein levels *in vitro*. A recent study focused on the genome-wide host transcriptional responses to CH-1a infection also showed increased expression of IL-10 in an *in vivo* infection model [32].

Our study showed that PRRSV infection significantly stimulated p38 phosphorylation (Figure 4a). And IκB was also gradually degraded in PRRSV-infected BMDMs (data not shown), which is consistent with a previous study [30]. In that report, the authors demonstrated that NF-κB pathway was activated in PRRSV-infected MARC-145 and PAMs through IκB degradation. Moreover, through the use of signal transduction pathway specific inhibitors, we further demonstrated that p38 MAPK and NF-κB pathways were involved in the up-regulation of IL-10 production in PRRSV-infected BMDMs (Figure 3). These results suggested that p38 MAPK and NF-κB signal transduction pathways played important roles in the induction of IL-10 during PRRSV infection. This is in accordance with previous reports, in which the authors showed that p38 MAPK pathway was essential for IL-10 production induced by LPS [26,33]. Another study also reported that IL-10 production stimulated by apoptotic cells was regulated at the transcription level in a p38 MAPK dependent manner [21]. Transcription factor NF-κB is also likely to be a candidate for the transactivation of the IL-10 gene, since the IL-10 promoter has nine putative NF-κB binding sites [34]. We also showed that p38 MAPK and STAT3 pathways were activated in GP5-transfected macrophages (Figure 7). It seems that p38 MAPK pathway is very important in IL-10 induction, since other viral proteins, for instance, extracellular HIV-Tat also induced IL-10 transcription in primary human monocytes through the activation of calmodulin/CaMK-II-dependent p38 MAPK [35,36]. The STAT3 transcription factor may also bind to an element in the IL-10 promoter and the use of a dominant negative form of STAT3 was able to decrease IL-10 transcription [37].

MAPK activation has also been shown to be required for the optimal replication of some viruses [38]. In this study, viral replication was not obviously affected when cells were treated with inhibitors of p38 (SB203580), ERK1/2 (PD98059), PI3K (LY294002), and NF-κB (BAY11-7082) pathways (Figure 2). A previous study showed that rhesus rotavirus replication was not significantly altered by the presence of p38 inhibitor, SB203580 at 10 μM in HT-29 cells or at 20 μM in MA104 cells [39]. Lee *et al.* (2012) showed that SB202190, another p38 inhibitor, down-regulated the viral gene expression only at high concentration (10 μM) [40]. Different p38 inhibitors might cause the differences in the effects of inhibitors on PRRSV replication in treated cells. Lee *et al.* (2010) also demonstrated that PD98059 had no effect on PRRSV replication in PAMs [41]. In contrast to our results, a recent report indicated that inhibition of PI3K/Akt by treatment with LY294002 at 25 μM prior to PRRSV infection reduced virus replication [42]. The different effects of PI3K inhibitor LY294002 on PRRSV replication might be due to the different concentrations used in their and our studies. In our report, we used no more than 10 μM.

GP5 is the most variable structural protein [43], and plays an important role in PRRSV pathogenesis. For instance, the existing of a decoy epitope in GP5 and glycan-shielding of the neutralization epitopes contributed to delayed and weak neutralizing antibody response [44,45]. GP5 was also suggested to be the apoptotic factor mediating the induction of apoptosis of uninfected bystander cells [46,47]. Recently, a study using chimeric viruses of a highly virulent strain vFL12 and an attenuated vaccine strain showed that NSP3-8 and ORF5 were the location of major virulence determinants [48]. Here, we showd that GP5 induced IL-10 production. Taken together, these results suggested that GP5 might play important roles in the pathogenesis of PRRSV infection. By using constructs truncated at different locations in GP5, the full-length of GP5 structure seems to be essential for IL-10 induction. Truncation of proteins will compromise their overall structure. Thus, more subtle changes could be introduced to map residues involved in specific functions (e.g. mutations) in the future.

Collectively, our results demonstrated that PRRSV strain CH-1a did up-regulate IL-10 production in macrophages. However, studies using vFL12, a virulent PRRSV strain, showed that there was no detectable level of IL-10 in the supernatant of PRRSV-infected macrophages and dendritic cells [25], and TNF-α was also poorly induced [49]. The authors suggested that the induction of IL-10 by several other PRRSV strains may be associated with their ability to induce pro-inflammatory cytokines during the acute phase of infection. However, IL-10 gene expression was up-regulated (1.9-fold) whereas TNF-α gene was only slightly up-regulated (1.5-fold) in Lelystad PRRSV strain-infected PAMs [20]. Another report demonstrated that exposure of BM-imDCs to PRRSV resulted in a significantly increased secretion of IL-10 but not TNF-α [50]. Interestingly, our lab found that Chinese highly pathogenic PRRSV (HP-PRRSV) infection failed to induce detectable IL-10 (unpublished data), but induced lower levels of TNF-α in PAMs [29]. Further studies need to be done to figure out whether IL-10 up-regulation is associated with virus induced immunosuppression as proposed or just associated with their ability to induce pro-inflammatory cytokines.

## Conclusions

In conclusion, our results showed that PRRSV strain CH-1a is one of those strains that can up-regulate IL-10 production in different types of swine macrophages (PAMs, BMDMs, and MDMs). Moreover, p38 MAPK signal transduction pathway played an important role in PRRSV induction of IL-10. This work may provide some insights into the molecular mechanisms of IL-10 regulation in swine macrophages during PRRSV infection.

## Methods

### Cell culture and virus preparation

MARC-145 cells, which is a PRRSV-permissive cell line sub-cloned from MA-104 cells (African green monkey kidney cells), were purchased from the China Institute of Veterinary Drug Control and maintained in Dulbecco’s minimum essential medium (DMEM) supplemented with 10% heat-inactivated FBS, 100U penicillin ml^-1^ and 0.1 mg streptomycin ml^-1^. PAMs were obtained by postmortem lung lavage of 8-week-old specific pathogen free (SPF) pigs, and maintained in RPMI 1640 supplemented with 10% FBS. PBMC were separated from peripheral blood and further differentiated into MDMs as described previously [51]. Bone marrow cells were flushed from femur and tibia and cultured in the same medium as MDMs. Non-adherent cells were removed on day 3. After 5 days of culture, differentiated macrophages (BMDMs) were used for further study. The PAM cell line 3D4/21 (CRL-2843) established by transformation of PAMs with SV40 large T antigen [52] was purchased from the American Type Culture Collection (ATCC) and maintained in RPMI 1640 medium supplemented with 10% FBS as described previously [31]. All the cells were maintained at 37°C with 5% CO_2._

CH-1a (the first PRRSV isolate in China), a North American genotype PRRSV strain, was propagated and titrated in MARC-145 cells. Inactivation of PRRSV was performed by UV irradiation of the virus suspension with 120 mJ cm^-2^ using a Bio-Link crosslinker (Vilber Lourmat). Virus inactivation was confirmed by inoculation of the UV-treated virus on MARC-145 cells followed by immunofluorescence assay against PRRSV N protein.

### Inhibition of signal transduction pathways

BMDMs were preincubated with DMSO, or various concentrations of PI3K inhibitor LY294002 (1 to 10 μM)), ERK1/2 MAPK inhibitor PD98059 (5 to 25 μM), p38 MAPK inhibitor SB203580 (1 to 10 μM), and NF-κB inhibitor BAY11-7082 (0.01 to 1 μM) (Enzo life Sciences) for 2 h and then infected with PRRSV at an MOI of 1 for 24 h. LPS (*E.coli* 055:B5) purchased from Sigma was used as a positive control for IL-10 induction. Cells were harvested and IL-10 mRNA levels were evaluated by real-time PCR. Secreted IL-10 levels were determined by ELISA. Virus titers in the cell supernatants were measured by a standard 50% tissue culture infective doses (TCID_50_) assay. The cytotoxicity of the inhibitors on BMDMs was determined by trypan blue exclusion dye staining.

### Construction and transfection of viral protein-encoding plasmids

Genes encoding viral proteins were amplified from CH-1a genome and cloned into pcDNA3.1-myc-his (N-terminal tagged) between *Kpn* I and *Bam*H I sites. Three clones of each constructs were sequenced and the clones of consensus sequence with the viral genome were used for further study. Truncated GP5 was amplified from pcDNA3.1-GP5 using primers listed in Table 1 and also inserted into pcDNA3.1-myc-his. The expression of these constructs was verified by western blot using rabbit anti-myc monoclonal antibody. PAMs and CRL-2843 cells were transfected using Amaxa Human Macrophage Nucleofector Kit in a Nucleofector II (Lonza) with program Y-001.

**Table 1:** Primer sequences

**Name**	**Sequence**
IL-10 F	CGGCGCTGTCATCAATTTCTG
IL-10 R	CCCCTCTCTTGGAGCTTGCTA
Cyclophilin F	AATGGCACTGGTGGCAAGTC
Cyclophilin R	GATGCCAGGACCCGTATGC
ORF5 F	GGGGTACCATGTTGGGGAAATGCT
N189 R	CGGGATCCCTATAAAGGGGTTGCCACGG
N125 R	CGGGATCCCTAAATGACGAAGCAAATCAACGC
N65 R	CGGGATCCCTACTCCACTGCCCAGTCAAAT
C126 F	GGGGTACCAGGCTTGCGAAGAACTGC
C66 F	GGGGTACCACTTTTGTCATCTTTCCCGTG
C30 F	GGGGTACCAACGCCAACAGCAACAGC
ORF5 R	CGGGATCCCTAGAGACGACCCCAT

Restriction enzyme sites are underlined.

### Real-time PCR

Total cellular RNA was isolated using a Total RNA Kit (OMEGA) and then on-column DNase I digestion was performed to remove any contaminating DNA. Reverse transcription was performed using M-MLV reverse transcriptase (Promega) with oligo (dT) 15 primer. IL-10 mRNA expression was analyzed by SYBR-green based real-time PCR using an ABI 7500 Real-Time PCR System. IL-10 mRNA copy numbers were normalized by comparing to housekeeping cyclophilin copy numbers and expressed relative to mock control. Primer sequences were listed in Table 1.

### ELISA

Cell supernatants of treated macrophages were centrifuged at 3,000 g for 5 min to remove cell debris and stored at −80°C until use. Secreted IL-10 in the cell supernatants were determined using commercial ELISA kits (R&D Systems) according to the manufacturer’s instructions.

### Western blot analysis

Whole-cell extracts from treated BMDMs or CRL-2843 cells were prepared as follows. Cells were washed twice with ice-cold PBS, lysed in 1% Triton X-100 lysis buffer (20 mM Tris–HCl, pH 7.4, 150 mM NaCl, 1 mM DTT, 1 mM EDTA, 10% glycerol, 1 mM DTT and 20 μM NaF ) for 15 min on ice. The lysates were centrifuged at 10,000 g for 20 min and the supernatant was aliquoted and stored at −80°C. Similar amounts of protein from each extract were separated by 10% sodium dodecyl sulfate-polyacrylamide gel electrophoresis (SDS-PAGE) and transferred to polyvinyl difluoride (PVDF) membranes (Millipore). After blocking for 1 h with blocking buffer (5% fat-free milk and 0.1% Tween-20 in PBS), the membranes were incubated for 2 h with the following primary antibodies diluted at 1:2,000: anti-phospho-p38 MAPK (no.9211), anti-p38 MAPK (no.9212), anti-phosphor-p44/42 MAPK(ERK1/2) (no.4370), anti-p44/42 MAPK(ERK1/2) (no.4695), anti-IκBα (no.9242) (Cell Signaling Technology), anti-phospho-STAT3, anti-STAT3 (Signalway Antibody), anti-myc, and anti-α-Tubulin (MBL) antibodies. HRP-conjugated anti-mouse IgG or anti-rabbit IgG (Santa Cruz Biotechnology) were used as secondary antibodies at a dilution of 1:5,000. The antibodies were visualized using ECL reagent (GE Healthcare) according to the manufacturer’s instructions.

### Luciferase reporter assays

pIL-10-luc plasmid was constructed by cloning porcine IL-10 promoter sequences into pGL3 basic vector (Promega) in our lab [53]. CRL-2843 cells were cotransfected with a mixture of pIL-10-luc plasmid, pRL-TK renilla luciferase plasmid, and viral structural protein-encoding plasmids or empty vector using Lipofectamine 2000 (Invitrogen). At 24 h post transfection, luciferase activities were determined with the Dual-Luciferase Reporter Assay System (Promega) according to the manufacturer’s instructions.

### Statistical analysis

All experiments were performed with at least three independent replicates. Data were analyzed using two-tailed *Student’s t-test* (paired). If the P value was less than 0.05, the difference was considered to be statistically significant.

## Competing interests

The authors declare that they have no competing interests.

## Authors' contributions

WHF designed the study and revised the manuscript. RQ and YF constructed viral structural protein-encoding plasmids and performed luciferase reporter assays. HXZ conducted virus isolation and helped in manuscript reviewing. JH and LHW performed all other assays presented, and drafted the manuscript. All authors read and approved the final manuscript.
